# Impact of Obesity on Pubertal Timing and Male Fertility

**DOI:** 10.3390/jcm14030783

**Published:** 2025-01-25

**Authors:** Valeria Calcaterra, Lara Tiranini, Vittoria Carlotta Magenes, Virginia Rossi, Laura Cucinella, Rossella Elena Nappi, Gianvincenzo Zuccotti

**Affiliations:** 1 Department of Internal Medicine and Therapeutics, University of Pavia, 27100 Pavia, Italy; 2 Pediatric Department, Buzzi Children’s Hospital, 20154 Milano, Italy; vittoria.magenes@unimi.it (V.C.M.); virginia.rossi@unimi.it (V.R.); gianvincenzo.zuccotti@unimi.it (G.Z.); 3 Department of Clinical, Surgical, Diagnostic and Pediatric Sciences, University of Pavia, 27100 Pavia, Italy; lara.tiranini01@universitadipavia.it (L.T.); laura.cucinella01@universitadipavia.it (L.C.); rossella.nappi@unipv.it (R.E.N.); 4 Research Center for Reproductive Medicine, Gynecological Endocrinology and Menopause, Fondazione IRCCS Policlinico San Matteo, 27100 Pavia, Italy; 5 Department of Biomedical and Clinical Science, University of Milano, 20157 Milano, Italy

**Keywords:** obesity, puberty, pubertal timing, fertility, infertility, children, adolescent, male, boys, reproductive function

## Abstract

Childhood obesity has profound effects on puberty in boys and girls, altering its timing, progression, and associated hormonal changes. Also, later male fertility could be impaired by childhood and pubertal obesity in light of the impact of inflammatory markers on semen quality. The aim of this narrative review is to explore the intricate relationship between childhood obesity and its impact on pubertal development and fertility, with a specific focus on boys. Such a relationship between obesity and pubertal timing in males is highly influenced by metabolic, hormonal, genetic, epigenetic, and environmental factors. While many studies suggest that obesity accelerates pubertal onset in boys, some studies do not confirm these findings, especially in cases of severe obesity. In fact, delayed puberty has also been reported in certain instances. Obesity influences fertility through different central and peripheral processes, including an altered endocrine milieu, inflammatory environment, and epigenetic modifications that alter semen quality and vitality, leading to subfertility or infertility. The early identification and management of potential issues associated with obesity are crucial for ensuring optimal reproductive health in adulthood. Further research is essential to clarify these associations and to develop targeted interventions aimed at preventing the negative health outcomes associated with obesity-related disruptions in puberty and fertility.

## 1. Introduction

Childhood obesity has become a global epidemic, with its prevalence rising significantly in both developed and developing countries over the last few decades. According to the World Health Organization (WHO), in 2020, over 39 million children under the age of five were classified as overweight or obese. Additionally, in 2016, approximately 340 million children and adolescents aged 5–19 were identified as being overweight or having obesity. Since 1975, the prevalence of obesity among children and adolescents has increased more than tenfold globally, rising from less than 1% to approximately 8–10% [1,2].

Puberty represents the physiological transition in which adolescents achieve sexual maturity and reproductive capability [3]. During this period, which occurs between 9 and 14 years of age in males, significant physical, hormonal, and psychological changes occur, culminating in the development of secondary sexual characteristics and the ability to reproduce, i.e., fertility. It is possible to distinguish three main phases, fetal and mini-puberty, adrenarche, and puberty, each with its own characteristics [3]. Gonadal function is regulated by hormones of the hypothalamic–pituitary–gonadal (HPG) axis. Hypothalamic neurons release gonadotropin-releasing hormone (GnRH), which enters the pituitary portal circulation, stimulating the production and secretion of gonadotropins. These gonadotropins, in turn, promote the synthesis and release of sex steroid hormones by the gonads [3,4].

Childhood obesity has profound effects on puberty in boys and girls, altering its timing, progression, and associated hormonal changes. These disruptions are driven by the interplay of excess adipose tissue, hormonal imbalances, and metabolic alterations, which can have short- and long-term health consequences [5,6,7,8,9].

Also, later fertility could be impaired by childhood and pubertal obesity, in light of the impact of inflammatory markers on semen quality [10,11,12,13].

Several mechanisms have been proposed to explain how obesity impacts pubertal onset and fertility in boys. These include genetic and epigenetic alterations, hormonal imbalances, environmental influences, nutritional deficiencies, and health-related comorbidities. Together, these factors highlight the complexity of the topic.

The aim of this narrative review is to explore the intricate relationship between childhood obesity and its impact on pubertal development and fertility, with a specific focus on boys. By examining the mechanisms through which obesity influences the timing and progression of puberty and fertility, as well as the hormonal and metabolic disruptions associated with it, this review seeks to elucidate the short- and long-term implications for reproductive health.

## 2. Methods

We conducted a narrative review [14,15] to investigate the relationship between childhood obesity and its effects on pubertal development and fertility in boys. We focused on the mechanisms by which obesity impacts the timing and progression of puberty and reproductive health, as well as the associated hormonal and metabolic disruptions. To achieve this, we performed an extensive literature search using the electronic database PubMed. The search included English language articles published within the last 25 years involving males. For the types of manuscript, original research articles, systematic and narrative reviews, meta-analyses, and longitudinal studies were included. Case reports, cases series, letters, and commentaries were excluded.

The search strategy incorporated specific keywords used individually and in combination as follows: child OR children OR childhood OR adolescent AND obesity OR obese OR overweight AND puberty OR pubertal timing OR delayed puberty OR early puberty OR minipuberty AND fertility OR infertility OR functional hypogonadism OR infertile males OR male infertility AND reproduction OR reproductive function.

Starting from a total of publications (n = 210), these were refined by screening the abstracts (n = 143) and performing detailed full-text evaluations of those relevant studies (n = 117) in which key findings related to search terms, inclusion criteria (sex), and study characteristics (type of article, manuscript language) were respected. We ended up with the studies critically analyzed in the present manuscript (n = 108). Additionally, the reference lists of all articles were reviewed to identify further relevant studies.

**Figure 1:**
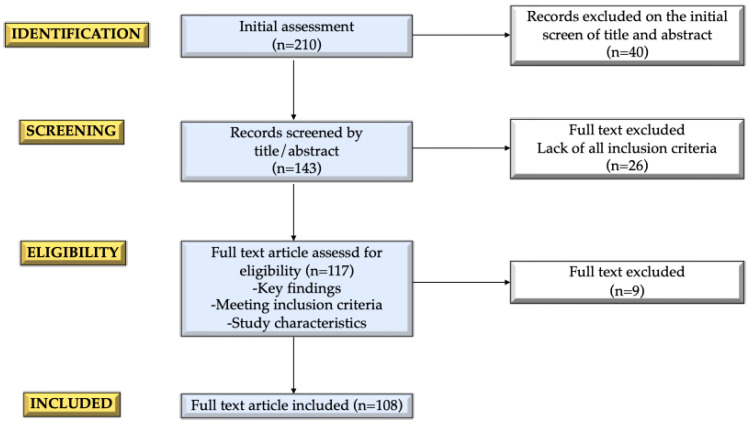
Flow diagram of the selection of the manuscripts.

The manuscript selection process is outlined in Figure 1. The draft of this review was collaboratively developed, reviewed, and approved by all co-authors to ensure the accuracy and comprehensiveness of the final analysis.

## 3. Pubertal Development and Reproductive Function in Males

Gonadal function is regulated by hormones of the HPG axis. GnRH, secreted by hypothalamic neurons in a pulsatile or continuous manner, enters the pituitary portal circulation [16]. It binds to GnRH receptors on gonadotropic cells in the anterior pituitary gland, stimulating the production and release of the gonadotropins’ luteinizing hormone (LH) and follicle-stimulating hormone (FSH). These hormones act on the gonads, driving the production of sex steroid hormones. The frequency and amplitude of GnRH pulses differentially influence the secretion levels of LH and FSH [16].

In males, LH stimulates Leydig cells in the testes to synthesize and release testosterone, with testosterone and 5-dihydrotestosterone (DHT) serving as the key bioactive androgens [17]. FSH, on the other hand, enhances the activity of Sertoli cells in the testes [18]. The HPG axis is regulated by feedback mechanisms from the gonads to the hypothalamus and pituitary gland. These mechanisms include both positive and negative feedback, where testosterone, estradiol, progesterone, and inhibins inhibit GnRH secretion, while LH and FSH contribute to positive feedback regulation [19].

Gonadal development and puberty can be divided into three main phases, namely fetal and mini-puberty, adrenarche, and puberty. Afterwards, the HPG axis is active throughout adulthood.

During the first phase, starting from the seventh week of gestation, the HPG axis is the leading player in fetal and child development. The activation of the HPG axis, which reaches its peak at 26 weeks of gestation, plays a key role in testicular descent, which is influenced by both anatomical and endocrine factors. The testes typically move from their initial position in the abdominal area, the urogenital crest, to the scrotum by the end of pregnancy. As early as the sixth week of gestation, Leydig cell precursors in the gonads begin producing testosterone, with levels peaking between 12 and 16 weeks of gestation [4]. Testosterone secretion is soon independent of the central HPG axis, which explains why males with severe GnRH or pituitary gonadotropin deficiencies in utero do not develop ambiguous genitalia. Testosterone serves two main functions in the male fetus: it promotes testicular descent and promotes penile development. Androgen insensitivity results in incomplete testicular descent, while penile and scrotal development, arising from the primitive urethral folds, is also influenced by the more potent metabolite DHT [4]. In fact, males with 5-α-reductase deficiency show penile hypoplasia and cryptorchidism [4,20]. Insulin-like factor 3 (INSL3), produced by Leydig cells in response to LH stimulation, is crucial in testicular descent by promoting the differentiation and growth of the gubernaculum, the genito-inguinal ligament [21,22]. Similarly, FSH facilitates the proliferation of Sertoli cells in the testes and increases the production of inhibin B and anti-Müllerian hormone (AMH) by these cells [4]. Furthermore, AMH is critical in fetal sex differentiation by promoting the regression of the Müllerian ducts [3,23].

Mini-puberty, first identified in the 1970s [24], occurs during the first six months of life in boys and is marked by a surge in gonadotropins and sex hormone secretion. This period is of fundamental importance for future reproductive capacity, as it triggers an increase in the number of Sertoli and germ cells in the testes, both of which are key determinants of future spermatogenesis, driven by the actions of LH and FSH [4,25]. As a result, testicular volume increases, peaking at around 4 to 5 months of age, with an approximate 40% growth compared to the size at birth. This growth then regresses by the age of 1 year, leaving the testicular volume slightly larger than it was at birth after the completion of mini-puberty [26]. Then, the HPG axis enters a quiescent state until the onset of puberty [27] and at this time, GnRH secretion is minimal with levels of LH, FSH, and sex steroids remaining undetectable [4].

After approximately the age of 6 years in girls and 7 years in boys, adrenarche begins [3,28,29,30]. Specifically, the term adrenarche refers to the time during puberty when the adrenal glands increase their production and secretion of weak adrenal androgens such as dehydroepiandrosterone (DHEA), its sulfate (DHEA-S), and androstenedione [3,28,29]. This process is related to the maturation of the innermost zone of the adrenal cortex, the zona reticularis, and it is independent of the HPG axis [3,4]. The term is also used to describe the development of axillary and pubic hair, although there are no clear and visible physical signs directly related to the initial increase in DHEA levels [3,28,29]. Pubarche specifically refers to the growth of pubic hair; however, it is often used interchangeably with adrenarche to include other clinical manifestations of androgen-stimulated maturation related to the development of sebaceous and apocrine glands, determining changes in body odor and skin, such as acne [3,4]. If these signs—together with the presence of serum adrenal androgen concentrations above the prepubertal reference range—present before 9 years in boys or 8 years in girls, the child is considered to have premature adrenarche [3,28,29,30].

This is a natural process that occurs in all healthy children, although the timing and extent of its manifestations vary between individuals [31]. For instance, studies show that the average age of pubic hair development slightly varies depending on race, sex, and ethnicity [31,32]. Recently, Augsburger et al. performed an interesting evaluation on adrenarche definition and consequences [33]. Indeed, the authors, after evaluating the literature of the past 5 years (including original articles, reviews, and meta-analyses), highlighted the fact that the molecular regulation and significance of adrenarche are still unknown, as this prepubertal event is characterized only descriptively. Moreover, the same authors stated that premature adrenarche should still be considered a diagnosis by exclusion exerting unclear long-term consequences [33]. Therefore, based on this recent revision of data, adrenarche is a mystery in many perspectives, as specific research into adrenal development and function is challenging for lack of experimental models because it occurs uniquely in humans [33].

In contrast, when hyperandrogenism is linked to an adrenal tumor, it typically presents in younger children, often during preschool years, and it progresses rapidly, displaying more pronounced signs of virilization compared to normal adrenarche [3]. Additionally, the early onset of adrenarche is more common in children born small for gestational age and in those who develop obesity thereafter [34]. It also tends to be more pronounced in individuals with a family history of conditions associated with hyperandrogenemia, such as polycystic ovary syndrome [3,34].

Eventually, puberty, which leads to complete physical and psychological maturation and the attainment of full reproductive capacity, occurs in males between the ages of 9 and 14 years [3,4,35]. It is marked by the development of male secondary sexual characteristics (virilization), and a key defining feature of puberty is the increase in testicular volume to 4 mL or more. The progressive increase in testicular size is a distinguishing characteristic of the various pubertal stages, as outlined by the Tanner staging system, which is a five-level observational method used to evaluate the progression of secondary sexual characteristics in males, such as genital development and pubic hair growth, ranging from the prepubertal phase (stage 1) to full maturity (stage 5) [36]. Initially, this growth is driven by an expansion of Sertoli cells and peritubular myoid cells. In later stages, testicular enlargement is predominantly due to the elongation and thickening of seminiferous tubules, resulting from the activation of spermatogenesis [4]. The final size of the testes is determined by the number of germ cells, which is directly linked to the population of Sertoli cells [37]. Each Sertoli cell facilitates the maturation of approximately 18 to 20 germ cells across different stages of spermatogenic development [4,38]. Serum inhibin B levels, secreted by Sertoli cells, serve as a reliable marker for spermatogenesis [39]. In males, LH stimulates Leydig cells in the testes to produce and secrete testosterone, paralleling the hormonal processes observed during mini-puberty. FSH, in conjunction with intratesticular testosterone acting through androgen receptors, stimulates Sertoli cells to support spermatogenesis [4].

The pubertal growth spurt, characterized by accelerated growth, also takes place during this phase [3,40]. This process is driven by the combined effects of sex hormones and the growth hormone (GH) insulin-like growth factor 1 (IGF-1) pathway. Testosterone supports longitudinal bone growth indirectly by being converted into estradiol, which enhances GH release and stimulates activity in the epiphyseal plates.

Another defining feature of puberty in males is the voice change, marked by a substantial lowering of pitch [40]. This transformation typically takes place during the transition between Tanner stages 3 and 4 [40].

Overall, male pubertal development is a complex, well-coordinated process governed by the reactivation of the HPG axis. The interaction between gonadotropins, sex hormones, and growth factors facilitates normal physical growth, the maturation of reproductive organs, and the achievement of full reproductive capability. Any disruptions in this progression, whether through delayed or early onset, can significantly affect growth, development, and fertility. Addressing these disturbances requires accurate diagnosis and targeted treatment, guided by a thorough understanding of the endocrine mechanisms involved.

**Figure 2:**
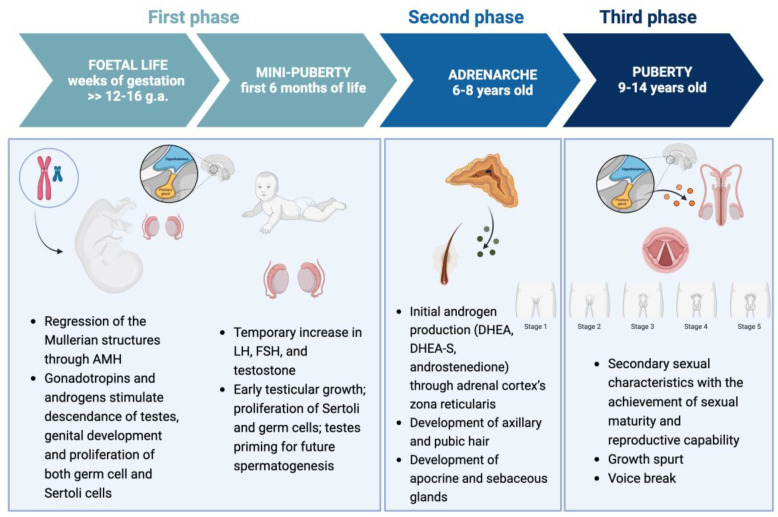
Phases of gonadal development and puberty (created by BioRender® accessed on 18 January 2025).

In Figure 2, the phases of puberty are schematized.

## 4. Impact of Obesity on Pubertal Timing in Males

### 4.1. Obesity and Pubertal Timing 

Obesity is a global epidemic with profound impacts on metabolic and developmental health, and one area of growing concern is its effect on pubertal timing [5,7,32,41,42]. A time trend toward earlier puberty in girls has been observed in many countries, but it is less established in boys [5,7,32,41,42]. In males, indeed, the evidence is more complex and less consistent, though various studies suggest that boys with obesity tend to experience earlier puberty, particularly gonadarche (testicular enlargement) [5,32,43].

Interestingly, Ahmed et al. performed a review on childhood obesity and puberty and evidenced that childhood obesity is associated not only with early puberty onset but also with greater variability in its progression [44]. Indeed, some boys experience early onset of certain pubertal features, while others show delays in specific markers, like voice breaking [44]. Coherently, Kaplowitz et al. further elaborated on the link between body fat and pubertal timing in girls and boys, highlighting that excessive adiposity influences puberty both directly, through hormones like leptin secreted by the adipose tissue, and indirectly, by affecting the release of sex hormones that accelerate physical maturation [45]. More recently, Brix et al. also reviewed the relationship between childhood obesity and the onset of puberty [43]. Their analysis of existing studies further confirmed that higher body mass index (BMI) in both boys and girls is linked to earlier pubertal onset, including earlier testicular development in boys and breast development in girls [43].

Consistently, Herman-Giddens et al. evaluated 4131 U.S. boys, aged 6–16 years, and analyzed the mean ages of beginning genital and pubic hair growth and early testicular volumes [46]. The authors found out that these characteristics were 6 months to 2 years earlier than in past studies, depending on the characteristic and race/ethnicity of the subjects [46]. Thus, they concluded that excessive body fat could accelerate the development of secondary sexual characteristics in boys, with an average pubertal onset of 11.9 years, earlier than normal weight peers [46]. Moreover, they underlined that the causes and health implications of this apparent shift in U.S. boys toward a lower age of onset for the development of secondary sexual characteristics require further exploration [46].

Interestingly, Brix et al. found out that an increase in BMI during childhood was associated with a significant advancement in pubertal onset in boys, suggesting that childhood obesity acts as a strong predictor of accelerated pubertal maturation [6,47]. In more detail, the authors analyzed 11,046 Danish children (boys and girls, aged 5 to 15 years) to investigate the relationship between childhood BMI and the timing of puberty, using a cohort design and a sibling-matched design [6,47].

The objective was to determine whether higher BMI in early childhood was associated with an earlier onset of pubertal milestones, namely pubarche and gonadarche in boys and thelarche in girls (the onset of breast development) [6,47]. The results indicated that both children with overweight and obese conditions experienced significantly earlier pubertal onset compared to normal weight peers [6,47]. This association was consistent across both sexes, with the influence of BMI on puberty being stronger in girls than in boys [6,47].

Busch et al., aiming to assess the association of male pubertal timing with age-specific BMI (zBMI) in boys with obesity, recruited 218 male subjects with obesity (zBMI > +2SD, with a median age at baseline of 10.8 years) as part of a prospective outpatient childhood obesity intervention program in Denmark between 2009 and 2017 [5]. The authors also evaluated 660 healthy boys as controls (−2SD < zBMI ≤ +2SD, 2006–2014) and evidenced that testicular volume ≥ 4 mL occurred significantly earlier in boys with obesity as compared to controls (*p* = 0.01) but any significant difference for either the timing of pubarche or the genital stage ≥ 2 (*p* = 0.06 and *p* = 0.94, respectively) was observed [5].

In addition, Deardorff et al. performed a longitudinal cohort study aiming to assess the relationship between childhood obesity and the timing of pubertal onset [48]. The authors evaluated 700 Mexican–American children aged 5–13 from the CHAMACOS cohort and measured BMI and pubertal milestones (as thelarche in girls and gonadarche in boys) [48]. Results showed that overweight or obese children had significantly earlier pubertal onset compared to their normal weight peers, with stronger associations observed in girls [48]. Thus, they confirmed that childhood obesity was a key factor in the earlier onset of puberty also in Mexican–American children [48].

More recently, Aghaee et al. conducted an observational study to investigate the sex- and race/ethnicity-specific relationships between childhood obesity and the timing of puberty in a multiethnic cohort of 129,824 adolescents born between 2003 and 2011 at Kaiser Permanente Northern California [32]. The study aimed to examine the associations between childhood obesity and the onset of breast development in girls, gonadarche in boys, and pubic hair development in both sexes [32].

The findings revealed a clear dose–response relationship: boys with severe obesity exhibited the highest risk for earlier gonadarche (hazard ratio = 1.23, 95% confidence interval: 1.15, 1.32) and pubarche (hazard ratio = 1.44, 95% confidence interval: 1.34, 1.55), while underweight boys experienced delayed puberty compared to their peers with normal body mass index [32].

While many studies support the link between obesity and earlier puberty in males, some findings are inconsistent. For example, Bygdell et al., aiming to investigate the relationship between childhood BMI and the timing of puberty in boys, analyzed a cohort of boys from Sweden, measuring their BMI and tracking the onset of puberty through testicular enlargement [49]. Their findings reveal an inverse link between childhood BMI and the timing of puberty in boys, which is evident in those with normal weight but absent in overweight individuals. The study concluded that BMI influences the timing of puberty in boys, but the effect is more pronounced in those who are within the normal weight range, suggesting that excess weight may alter the usual developmental pathways [49].

Moreover, Lee et al. conducted a longitudinal prospective study, tracking 401 boys (aged 5–12 years) from the U.S., and they measured BMI alongside markers of pubertal initiation, including genital development and pubic hair growth [9]. The results indicated that boys with higher BMI had a significantly delayed onset of puberty compared to normal weight peers [9]. Thus, the researchers concluded that increased body weight in early childhood may be associated with delayed pubertal initiation in boys [9].

The same authors also carried out a cross-sectional study analyzing data from 1601 boys aged 6–16 years across multiple U.S. pediatric practices [8]. The researchers measured BMI and monitored pubertal markers such as testicular enlargement and pubic hair growth [8]. Their results showed that boys with obesity tended to experience delayed puberty compared to overweight boys, who instead had earlier pubertal onset than their normal weight peers [8]. Thus, they confirmed that obesity is linked to delayed puberty in boys, while overweight boys may experience earlier puberty, highlighting the complex relationship between body weight and pubertal timing [8].

In addition, Chung et al. examined how obesity affected growth and pubertal development, underlining that excessive adiposity often leads to an earlier onset of puberty in girls but may cause inconsistent effects in boys, including delayed puberty in some cases [50]. According to the authors, the delayed pubertal onset could be due to the disrupted function of the HPG axis related to insulin resistance and hyperinsulinemia in boys with obesity [50].

Moreover, Mohsenipour et al. performed a cross-sectional study, investigating 168 children with obesity from Tehran, Iran, from March 2018 to February 2019, hypothesizing that puberty onset was disturbed as the children gained more weight [51]. The authors showed no difference between males and females regarding early puberty (*p* = 0.098), but delayed puberty was significantly higher among males with obesity than among girls with obesity (*p* = 0.029) [51].

Discrepancies may arise due to methodological differences in measuring puberty (e.g., testicular volume vs. pubic hair development), differences in study populations, or unmeasured factors such as diet and physical activity [5,32,43,46].

It is worthwhile to underline that the long-term consequences of early puberty in boys are concerning. Early pubertal onset is associated with an increased risk of metabolic syndrome, cardiovascular diseases, type 2 diabetes, and shorter adult stature due to premature closure of the growth plates [5,49]. Therefore, managing childhood obesity is crucial not only for immediate health benefits but also for mitigating the long-term risks associated.

In Table 1, the main articles included in this review on the relationship between obesity and pubertal timing in males are summarized.

### 4.2. Mechanisms Linking Obesity and Puberty

Several mechanisms have been proposed to explain how obesity influences pubertal onset in boys.

One of the most studied pathways involves leptin, an adipokine produced by adipose tissue, which increases in proportion to body fat. Leptin regulates energy homeostasis and reproductive function, acting on the hypothalamus to influence the secretion of GnRH [5,54]. Elevated leptin levels in boys with obesity were shown to prematurely activate the HPG axis, leading to earlier gonadarche and other pubertal milestones [52,53]. Interestingly, the same adipokine, although more frequently associated with earlier puberty, has been associated also with delayed puberty upon disruption of the normal pulsatile secretion of GnRH [44].

Insulin and insulin resistance (IR) play a central role in the complex relationship between obesity and puberty, significantly influencing gonadal function through multiple pathways [5,54]. Obesity-related hyperinsulinemia not only increases androgen production and alters the bioavailability of sex hormones but also directly impacts the HPG axis. In males, one mechanism involves insulin’s role in the maturation of Sertoli cells in the testes, which is essential for testicular development and spermatogenesis [54]. Hyperinsulinemia and impaired insulin signaling can disrupt this maturation process, delaying puberty. The link between reduced serum testosterone levels and decreased insulin sensitivity highlights the early onset of gonadal dysfunction driven by IR. While this association is well-documented in adult males, it is increasingly observed in adolescents, suggesting that metabolic disturbances can impair reproductive health from a younger age [55]. Furthermore, while in males, insulin resistance is typically linked to lower testosterone levels, in females, hyperinsulinemia typically leads to hyperandrogenism [55].

The hormonal fluctuations of puberty, marked by rising levels of growth hormone and IGF-1, add another layer of complexity to insulin sensitivity during this critical stage. Adolescents, especially those with type 1 diabetes mellitus, frequently experience increased insulin demands, reflecting a temporary state of insulin resistance even in the absence of obesity [56]. When combined with excess adiposity, this physiological insulin resistance intensifies, contributing to sustained hyperinsulinemia and an elevated risk of gonadal dysfunction [56].

This complex interplay between metabolic health and pubertal development underscores the importance of addressing early on obesity and IR to prevent potential disruptions in gonadal function and reproductive health [47]. However, this area needs to be further investigated to better understand the mechanisms linking obesity, insulin resistance, and puberty.

Adipose tissue contains aromatase, an enzyme that converts androgens into estrogens; increased aromatase activity in boys with obesity may lead to higher circulating estrogen levels, which could contribute to the earlier development of secondary sexual characteristics [45]. Interestingly, higher estradiol concentrations have been measured in children with obesity compared to those with normal weight. However, the precise effect of this process on pubertal development remains undefined, and the literature presents conflicting findings on this issue [54]. Indeed, as obesity increases the incidence of hypogonadism in men and hypogonadism in turn plays a role in obesity, it has been hypothesized that men have higher estrogen levels and that increased estrogen provides feedback to the hypothalamic–pituitary–testicular axis, reducing gonadotropin secretion and testosterone. This concept has been questioned but never disproven [57].

Nutritional, hormonal, and environmental factors during critical developmental periods (such as the periconceptional, prenatal, and early postnatal stages) play a crucial role in pubertal maturation, gonadotropic function, and adult fertility. Early-life exposure to adverse conditions, including unhealthy lifestyles, hormonal imbalances, maternal stress, and endocrine disruptors, can disrupt reproductive development through mechanisms like epigenetic changes and alterations in hypothalamic regulators such as the Kiss1 system [58]. Research in both animal and human models has linked maternal and paternal obesity and high-fat diets or even undernutrition during the periconceptional period to reproductive abnormalities in the offspring of both sexes [58,59].

Epigenetic processes, particularly DNA methylation, appear critical in this context [58,60,61]. For example, a recent pilot study by Ponce et al. [61] investigated whether prepubertal androgen exposure was associated with differential methylation profiles in pubertal girls. The findings revealed distinct methylation patterns between girls with and without biochemical premature adrenarche. However, as this study focused only on female participants and had a limited scope, further research is needed to draw definitive conclusions, especially regarding males.

Genetic factors also contribute to the relationship between obesity and puberty. Bell et al. used Mendelian randomization to show that genetic variants associated with higher BMI were linked to earlier puberty onset, suggesting a shared genetic predisposition for both obesity and early pubertal maturation [52]. Similarly, Cousminer et al. identified genetic loci associated with both BMI and pubertal timing, further supporting the role of shared genetic pathways [53].

In summary, a combination of epigenetic modifications, genetic predisposition, early-life stress, and nutritional changes may accelerate biological aging and influence the timing of puberty through changes in gene expression. Further research is essential to clarify these mechanisms and address knowledge gaps, particularly in the male population.

Additionally, exposure to endocrine-disrupting chemicals (EDCs), which are prevalent in industrialized societies, can interfere with hormonal regulation and may contribute to both obesity and earlier puberty [54,62].

Another area of interest is the gut microbiota, which is influenced by diet and obesity. Research suggests that obesity-related changes in gut microbiota may promote earlier pubertal onset by modulating hormonal signaling and inflammation [53].

## 5. Obesity and Male Fertility

In recent decades, the human fertility rate has declined concurrently to an increasing incidence of obesity and metabolic diseases [63]. Indeed, obesity may influence male fertility through various mechanisms that impair spermatogenesis, including alterations of reproductive hormones, IR, chronic low-grade inflammation, altered adipokines, and epigenetics [64,65,66]. Even if childhood and puberty pave the way for later fertility, only a few studies, mainly on animal models, have focused on the impact of childhood and pubertal obesity on adult reproductive function [63]. In a retrospective study involving 268 children and adolescents followed for weight control, peri-pubertal BMI was found to be associated with testicular volume, which in turn was correlated with sperm production. Boys with normal weight showed significantly higher testicular volume compared with controls with obesity and overweight conditions. This association was not present in other age ranges, supporting the importance of weight control throughout puberty for maintaining testicular function later in life [67]. Although specific physio-pathologic pathways have not been explored in light of childhood obesity, the main mechanisms relevant for the modulation of fertility in men with obesity are reported in the following paragraphs.

### 5.1. Functional Hypogonadism

Obesity in adult males alters the HPG axis, causing a condition of hyperestrogenic hypogonadotropic hypoandrogenemia [65]. Indeed, excess adiposity increases the peripheral aromatization of testosterone into 17β-estradiol [68,69]. Furthermore, increased estrogen alters the neuroendocrine regulation of the HPG axis through a decreased production of kisspeptin that blunts hypothalamic GnRH pulsatile release and subsequent pituitary FSH and LH secretion [68,69]. The overall result is lower circulating testosterone, which has a pivotal role in male fertility. Indeed, testosterone regulates Sertoli cell differentiation and proliferation, maintains the blood–testis barrier through the induction of Sertoli cell tight junctions, and promotes the expression of FSH-induced genes involved in spermatogenesis [70,71]. Moreover, lower testosterone levels increase lipogenesis with a further accumulation of adiposity, maintaining a vicious circle; thus, the relation of obesity with hypogonadism in males can be considered bidirectional [72,73]. At the same time, concurrent hyperestrogenism impairs Sertoli cell metabolism and alters the blood–testis barrier, contributing to impaired male fertility [64,74].

As noted earlier, the relationship between hyperestrogenism and obesity remains a topic of ongoing debate. Indeed, Pospisilova et al. performed an interesting study evaluating more than 200 men, with a broad range of BMIs (from 18 to 39) [57]. Researchers evaluated anthropometric data, and levels of serum total testosterone, free testosterone, estradiol, SHBG, LH, and FSH were measured using an immunoradiometric assay. Then, the authors divided the subjects in subgroups according to BMI (normal weight men, overweight, and obese males) and compared hormone levels between groups [57]. The result was that the differences in estradiol levels between the groups were not significant, strengthening the idea that estrogen production in men with overweight conditions and obesity does not significantly influence endocrine testicular function [57].

Kley et al. [75] demonstrated that in a large number of individuals with obesity, plasma testosterone levels decreased significantly, while free testosterone levels remained stable. Concurrently, estrone and estradiol levels showed significant increases, with a notable rise in free estradiol, suggesting a potential link between increased fat tissue and enhanced estrogen production. However, more recent findings by Stárka et al. [76], comparing hormone levels among men with varying BMI levels, revealed that although SHBG and overall testosterone levels declined with increasing BMI, there were no significant changes in free androgens, estradiol, or gonadotropins. These findings challenge earlier assumptions about hyperestrogenism in obesity.

Furthermore, obesity is closely associated with infertility through mechanisms involving disruptions in fatty acid metabolism, lipotoxicity, and reduced vitamin D levels. Low vitamin D levels, commonly observed in obese individuals, are known to adversely affect reproductive function in both men and women [77]. Engin et al. [78] emphasized that lipotoxicity, resulting from an excess of free fatty acids (FFAs) due to hypertrophic and dysfunctional adipocytes, led to systemic inflammation, endoplasmic reticulum (ER) stress, and cellular damage in non-adipose tissues.

In animal studies, lipotoxicity has also been shown to affect reproductive tissues. For instance, in obese female mice, it caused mitochondrial dysfunction, apoptosis, increased ER stress markers, and decreased fertility rates [79]. Similarly, in male mice, high-fat diets (HFDs) resulted in lipid accumulation in the testicular interstitium, oxidative stress, and disrupted blood–testis barrier integrity, all of which significantly impaired fertility potential. Notably, switching from an HFD to a normal diet has been shown to reverse these effects and restore reproductive function [80]. Furthermore, a study by Luo et al. [81] in a rat model highlighted that Sertoli cell dysfunction in obese males, driven by disrupted fatty acid oxidation and impaired energy metabolism, further compromised spermatogenesis and sperm quality.

These findings underscore the pivotal role of lipotoxicity in both male and female infertility linked to obesity. Promising therapeutic approaches, including dietary modifications, increased intake of eicosapentaenoic and docosahexaenoic acids, and pharmacological treatments such as metformin and GLP-1 receptor agonists, may help alleviate lipotoxicity and potentially improve reproductive outcomes in obese individuals [78].

Collectively, larger randomized control trials are needed to better characterize the implication of overweight conditions on male reproductive potential [64,82].

### 5.2. Insulin Resistance and Hyperglycemia

Insulin-resistance has been observed in males with unexplained infertility [10]. Its effect on the HPG axis is mediated by increased inflammation and a decreased liver production of sex hormone-binding globulin (SHBG) that leads to increased free estrogens [11,83]. It was observed that IR is associated with a lower sperm motility and an impaired capacitation and when combined with obesity, it may reduce semen volume and sperm count [84,85]. Moreover, at the molecular level, IR interferes with the FSH signaling through shared intracellular pathways, thus leading to altered FSH-induced Sertoli cell proliferation and spermatogenesis [64,86]. Accordingly, it was observed that infertility treatment with FSH had reduced efficacy in men with IR [12]. Hyperglycemia, as observed in type 1 and 2 diabetes mellitus, has been shown to negatively influence male reproductive capacity through various intracellular signaling pathways and pro-inflammatory mechanisms, resulting in impaired steroidogenesis and spermatogenesis [11,87,88,89]. Interestingly, Maresch et al. recently showed three major biochemical pathways implicated in the pathogenesis of hyperglycemia-induced testis and epididymis damage, namely the advanced glycation end product formation pathway, the diacylglycerol–protein kinase C pathway (PKC), and the polyol pathway [87]. The pathways shown to be more involved in reproductive impairment (in a type 1 diabetes mouse model) seem to be the diacylglycerol–PKC and the polyol pathway [87]. This may provide therapeutic opportunities in the treatment of diabetic male reproductive dysfunction, but the implications in humans and possible therapeutic approaches have to be confirmed by further studies [87,88].

### 5.3. Chronic Inflammation

Obesity is associated with chronic low-grade inflammation that may cause subfertility, as demonstrated in groups of men with obesity showing high levels of inflammatory markers and reduced serum testosterone [90,91]. Adipocytes produce several pro-inflammatory cytokines, such as tumor necrosis factor (TNF)-α and interleukins (ILs), which also impact spermatogenesis [64]. Some cytokines, as TNF-α and IL-6, alter tight junctions between Sertoli cells, influence Leydig cell production of testosterone, and induce the chemoattraction of immune cells such as macrophages and neutrophils in the epididymis, thus altering sperm maturation [65,92]. Indeed, this inflammatory microenvironment at the testicular level is reflected by an altered sperm proteome in men with obesity compared with healthy adults [93]. At the same time, inflammatory cytokines also manifest a central action on the HPG axis through the inhibition of GnRH and LH secretion, thus leading to low testosterone production [64,94].

Obesity increases the production of reactive oxygen species (ROS), which may affect sperm quality [73,95]. Indeed, ROS can damage sperm membrane and sperm mitochondrial DNA, thus compromising spermatozoa [95]. Moreover, increased ROS in the endothelial cells of corpora cavernosa inhibit nitric oxide production and, therefore, vasodilatation, thus contributing to erectile dysfunction [96].

C-reactive protein (CRP), an important inflammatory marker shown to be positively correlated with obesity, may also impact male fertility, especially in obese individuals [90,91,97]. As previously mentioned, similarly to CRP, other pro-inflammatory cytokines, such as TNF-α and interleukins, can influence the HPG axis. A multi-ethnic study by Osmancevic [98] examined the roles of IL-6 and CRP in relation to testosterone and SHBG in men. An inverse association was observed between high-sensitivity CRP levels and both testosterone and SHBG. Similarly, IL-6 levels were inversely associated with bioavailable testosterone while showing a positive association with SHBG. These findings underscore the complexity of interactions between inflammatory markers and SHBG, which seem to act independently. No associations were found between the soluble TNF receptor and endogenous sex hormones. This complexity highlights the need for further research to fully elucidate the role of inflammatory cytokines, including IL-6 and TNF-α, in modulating testosterone levels and SHBG interactions.

However, further studies are needed to clear out the direct relation between the two entities [99].

### 5.4. Adipokines

Adipose tissue is an endocrine organ secreting several peptides, namely adipokines, which exert cardiometabolic, immunomodulating, and reproductive functions [100,101,102]. In individuals with obesity, altered adipokines may contribute to poor fertility outcomes through various direct and indirect mechanisms, including an altered expression of steroidogenic genes [100,101,102].

Leptin mediates anorexigenic signals and modulates the HPG axis by promoting kisspeptin activity on GnRH neurons [103,104]. Childhood obesity, hypogonadotropic hypogonadism, and delayed puberty have been observed in congenital leptin deficiency [105]. Interestingly, hyperleptinemia was observed also in individuals with obesity, thus hypothesizing a condition of “leptin resistance”, meaning an abnormal increase in leptin levels without a decrease in appetite or an increase in energy expenditure [106]. In this context, an obesity-associated increase in leptin may influence reproductive function through various mechanisms, such as an inhibitory effect on the kisspeptin–GnRH pathway [107], an altered nutritional support of germ cells due to the impaired metabolism of Sertoli cells that express leptin-receptors [108], an impaired testosterone production in Leydig cells, and an increased oxidative stress with mitochondrial dysfunction in spermatozoa [102].

Adiponectin has anti-inflammatory properties and improves insulin sensitivity [109]. Its serum concentration is inversely proportional to visceral adiposity and BMI [110,111], and it was observed that adiponectin levels in semen were lower in men who were overweight and obese compared to normal weight controls [112]. Adiponectin was positively correlated to sperm count and concentration and normal spermatozoa morphology [112].

Chemerin inhibits insulin signaling as opposed to adiponectin [102]. Its levels correlate directly to BMI and inversely to testosterone levels [112]. Chemerin and its receptors were found in testes and appeared to have adverse effects on testicular steroidogenesis [113,114].

Resistin is an adipokine implicated in the development of insulin resistance and pro-inflammatory environment [110]. Its impact on male fertility is controversial; however, some evidence showed a negative correlation with sperm motility and vitality [115].

### 5.5. Sirtuins

Sirtuins are proteins with deacetylase enzymatic activity that regulates cellular metabolism [102]. Among sirtuins, SIRT1 was decreased in individuals with obesity, thus promoting adipocyte differentiation [116,117]. In males, sirtuins appeared to positively modulate spermatogenesis through the testicular regulation of various phases of sperm maturation, as glycosylation and lactate production in Sertoli cells, glycolysis, fatty acid oxidation, protection from oxidative stress, and chromatin remodeling [64,118]. Moreover, sirtuins acted centrally by enhancing GnRH secretion [119,120].

### 5.6. Irisin

Irisin is an adipo-myokine primarily produced by striated muscle under physical exercise, which endorses different metabolic roles by inducting white fat browning, regulating glucose metabolism, and improving insulin sensitivity [121]. Its properties were also recently investigated in the reproductive field, with contrasting results in an animal model [64]. One study observed a negative correlation between testosterone levels and irisin in human males with obesity [122].

### 5.7. Gut Hormones

Hormones produced by the gastrointestinal tract are involved in various metabolic processes and recent evidence showed an impact also on reproductive function [64].

Ghrelin is a peptide secreted by the gastric fundus cells during fasting states and has an orexigenic role through a hypothalamic regulation [64]. Its levels are inversely correlated to BMI [123]. Ghrelin has a protective role in male fertility. Indeed, it reduced the effects of heat stress-induced testicular damage and modulates Sertoli cell glycolytic metabolism and the nutritional support of germ cells according to the individual energetic state [124,125]. Low levels of ghrelin have been associated with lower semen volume, sperm motility, and morphology [65].

Glucagon-like peptide-1 (GLP-1) is an incretin hormone that regulates pancreatic insulin secretion and glucagon release according to nutrient ingestion and glucose serum levels [64]. It also plays a role in male reproduction, promoting sperm motility and viability [64]. Indeed, GLP-1 receptors were expressed both in Sertoli cells, where GLP-1 enhanced nutritional support and antioxidant protection for germ cells, and in Leydig cells, where it modulated steroidogenesis and sperm glucose metabolism [126,127]. Accordingly, an interventional study revealed that either or both GLP-1 analog liraglutide and physical exercise improved sperm concentration and sperm count in patients with obesity [128].

### 5.8. Gut Microbiome

An altered gut microbiome, as observed in metabolic diseases and systemic inflammation, can influence androgen production, the blood–testicular barrier, and spermatogenesis. However, to date, studies are limited and mainly carried out in animal models [64,129].

### 5.9. Sperm Transcriptome and Epigenetic Modifications

Human spermatozoa contain various types of RNA (messenger RNA [mRNA], micro RNA [miRNA], long non-coding RNA [lncRNA], etc.) that contribute to spermatogenesis, fertilization, and embryo development [130]. Obesity may alter the expression of RNA in spermatozoa and seminal plasma, negatively affecting male fertility [64]. Indeed, it was observed that some sperm RNA and DNA methylation patterns were significantly different in males with obesity compared to controls, and subsequent weight loss induced by bariatric surgery modified sperm DNA methylation [131]. Moreover, recent evidence showed that patients with obesity had reduced sperm telomere length compared to normal weight controls, with an associated increase in the sperm DNA fragmentation index and ROS production [13]. Histone acetylation is a protective mechanism for sperm DNA integrity, and it was observed to be impaired in individuals with obesity, thus resulting in increased DNA damage [132]. Another study showed that men with obesity had increased levels of miRNA-122 and mi-RNA-155 in seminal plasma in comparison to controls [133]. These miRNAs were already known to correlate with inflammation [134] and altered iron homeostasis [135]. Growing evidence suggests that parental obesity is transferred to the offspring through epigenetic modifications [136]. Considering that epigenetic transfer involves reversible alterations without DNA mutations, the removal of triggering factors, such as obesity, can switch off negative epigenetic marks, thereby allowing for a positive transgenerational influence on subsequent generations [131,137].

Faienza et al. [138] published a review on the interplay between genetic, epigenetic, and environmental factors in regulating pubertal timing. Genetic and epigenetic modifications primarily impacted genes encoding kisspeptin, GnRH, LH, FSH, and their corresponding receptors, key components of the HPG axis. Epigenetically modulated genes, such as Makorin ring finger 3 (MKRN3) and Delta-like 1 homologue (DLK1), played central roles in pubertal development, acting as repressors and activators, respectively. Paternally inherited DLK1 defects have been linked to CPP and metabolic issues like obesity, early glucose intolerance, type 2 diabetes, and hyperlipidemia. DLK1 mutations were also associated with polycystic ovary syndrome (PCOS) and infertility, highlighting the connection between metabolism and reproduction. MKRN3 delayed puberty by epigenetically silencing GNRH1 through the poly-ubiquitination of MBD3, preventing its interaction with TET2 (a DNA demethylase) and the GNRH1 promoter. This process inhibits puberty initiation, reinforcing the role of epigenetic regulation in pubertal timing [138].

In Table 2, the main articles included in this review on the relationship between obesity and male fertility are summarized.

### 5.10. Role of Weight Loss in Improving Fertility Outcomes

Several studies in adult males with obesity have demonstrated that weight loss can overthrow endocrine disturbances, leading to increased serum testosterone levels and improved sexual function [82]. Ballestrella et al. [139] reported an improvement in sex hormone levels, as well as occasional enhancements in sexual function and semen parameters.

Similarly, Collins et al. [140] investigated the short- and long-term effects of weight loss through diet, observing sustained increases in total and free testosterone levels during both the weight loss and maintenance phases. These findings underscore the significance of weight loss in correcting hormonal imbalances associated with obesity, reinforcing the importance of lifestyle modifications as a first-line treatment for hypogonadism in this patient population.

The impact of weight loss on semen parameters and quality is less extensively studied. A cohort study by Håkonsen et al. [141] assessed obese men participating in a 14-week weight loss program, revealing an inverse relationship between BMI and sperm concentration, total sperm count, sperm morphology, testosterone, and inhibin-B, along with a positive association with estradiol levels. Weight loss was linked to increases in total sperm count, semen volume, testosterone, SHBG, and AMH. Men who experienced the greatest weight loss showed significant improvements in total sperm count and normal sperm morphology. However, the study concluded that these improvements might not solely result from weight loss but could also reflect broader lifestyle changes.

In addition to weight loss, metabolic changes associated with dietary interventions may also influence hormonal regulation. For example, a three-week ketogenic diet (KD) has been shown to increase SHBG levels in obese men and women, accompanied by reductions in the free androgen index (FAI) and free estradiol index (FEI) in both sexes [142]. These changes suggest an interplay between β-hydroxybutyrate (β-OHB), a metabolite produced during ketosis, and the HPG axis. While the clinical significance of these hormonal alterations requires further investigation, they highlight the potential of KD-induced metabolic changes in modulating reproductive function.

## 6. Limitations

We acknowledge several limitations in this review. To begin with, it is a narrative review, which provides a non-systematic summary and analysis of the existing literature on a given subject [14,15]. The lack of formal guidelines in conducting narrative reviews can introduce selection biases and often results in qualitative rather than quantitative syntheses. For instance, our review was limited to articles available on PubMed, potentially overlooking relevant studies indexed in other databases or search engines.

Another notable limitation is the absence of a meta-analysis, which could have strengthened the quantitative assessment of the relationship between obesity and male fertility. Although this was not the primary objective of our work, we acknowledge that performing a meta-analysis focusing on the original studies and excluding review articles would have improved the overall impact and provided stronger evidence. We recognize that this is a valuable pathway for future research.

An additional limitation is the scarcity of long-term monitoring in existing studies. Evidence regarding the long-term complications remains insufficient. Conducting more longitudinal research is essential to explore how variations in puberty impact fertility in adulthood.

Furthermore, studies evaluating the long-term effects of obesity and pubertal timing on male fertility are limited by confounding factors such as lifestyle and environmental influences, which complicate the establishment of causal relationships between obesity and fertility in males.

Finally, the use of different cutoffs to define obesity limits the ability to compare its impact on puberty and fertility based on the severity of obesity. Furthermore, studies assessing pubertal development have employed highly heterogeneous parameters. Specifically, the lack of uniformity in defining the age of adrenarche and the frequent interchangeable use of the term pubarche with adrenarche make direct comparisons challenging.

Conducting well-designed longitudinal studies with larger more described populations will be essential to advance our understanding of the impact of obesity on pubertal timing and male fertility.

## 7. Conclusions

Puberty in boys is a multifaceted process that is essential for achieving fertility. The relationship between obesity and pubertal timing in males is intricate, being influenced by metabolic, hormonal, genetic, epigenetic, and environmental factors. While many studies suggest that obesity accelerates pubertal onset in boys—particularly with regard to testicular enlargement and other pubertal markers—some studies do not confirm these findings, especially in cases of severe obesity. In fact, delayed puberty has also been reported in certain instances. Also, fertility is influenced by obesity through different central and peripheral processes, including altered endocrine milieu, inflammatory environment, and epigenetic modifications that alter semen quality and vitality, leading to subfertility or infertility.

The rarly identification and management of potential issues are crucial for ensuring optimal reproductive health in adulthood. Further research is essential to clarify these associations and to develop targeted interventions aimed at preventing the negative health outcomes associated with obesity-related disruptions in puberty and fertility.

## Figures and Tables

**Table 1:** The main articles on the relationship between obesity and pubertal timing in males.

Reference	Type of Study	Study Population	Main Results	Conclusion
Aghaee et al. (2022) [32]	Observational cohort study	8500 boys and girls aged 6–18 (mean age: 12.5 years)	Childhood obesity was linked to earlier pubertal onset, particularly in specific ethnic groups (*p* < 0.01).	Obesity advances pubertal timing with differences by race/ethnicity
Ahmed et al. (2009) [44]	Review	Various studies on childhood obesity and puberty (no specific population size)	Obesity was linked to both earlier and more variable pubertal timing (*p* < 0.05 in selected studies).	Obesity may lead to earlier puberty, but the progression can vary
Bell et al. (2018) [52]	Mendelian randomization	264,000 boys and girls from UK Biobank cohort (mean age: 8 years)	Higher childhood BMI causally linked to earlier pubertal onset in both boys and girls (*p* < 0.001)	Childhood obesity may directly cause earlier pubertal onset
Brix et al. (2019) [6]	Population-based cohort	11,046 Danish boys and girls aged 6–18 years	Higher BMI was associated with earlier puberty onset in both sexes (*p* < 0.01)	BMI is a key predictor of pubertal onset timing
Brix et al. (2020) [47]	Cohort and sibling-matched study	11,046 boys and girls from a birth cohort (aged 5–15 years)	Childhood overweight and obesity were associated with earlier puberty in both boys and girls (*p* < 0.001)	Sibling-matched analyses confirm the association between obesity and earlier puberty
Brix et al. (2021) [43]	Review	Review of studies on childhood obesity and pubertal timing (no specific population size)	Both boys and girls with obesity experienced earlier pubertal timing, with significant associations found across different studies (*p* < 0.05)	Obesity in both boys and girls is associated with earlier pubertal timing, highlighting the impact of childhood BMI on development
Bygdell et al. (2018) [49]	Cohort study	Swedish cohort of boys aged 6–18 years	Higher BMI was associated with later pubertal onset in normal weight boys but not in overweight boys (*p* < 0.05)	BMI influences pubertal timing in boys with a stronger effect in normal weight individuals
Busch et al. (2020) [5]	Longitudinal cohort study	218 boys with obesity (mean age: 10.8 years) and 660 controls in Denmark	Boys with obesity experienced earlier testicular enlargement compared to controls (*p* = 0.01)	Obesity is associated with earlier pubertal onset in boys
Calcaterra et al. (2023) [7]	Review	Review of various studies on childhood obesity, high-fat diets, and central precocious puberty (no specific sample size)	Consistent associations between childhood obesity, high-fat diet, and earlier onset of central precocious puberty with obesity being a key risk factor for earlier pubertal timing (*p* < 0.01 across multiple studies)	Childhood obesity and high-fat diet are significant risk factors for central precocious puberty with implications for early pubertal development in children
Calcaterra et al. (2024) [7]	Review	Various studies on food contaminants and their impact on childhood development (no specific population size)	Phthalates and bisphenol exposure in foods were linked to precocious puberty and early-onset obesity (*p* < 0.05 across multiple studies)	Phthalates and bisphenol exposure through food may contribute to earlier pubertal onset and obesity in children, emphasizing the need for dietary interventions
Chung et al. (2017) [50]	Review	Various studies on childhood obesity (no specific sample size)	Obesity in boys often delayed puberty due to insulin resistance and hyperinsulinemia, which disrupt normal HPG axis function (*p* < 0.05)	Obesity may delay puberty in boys due to metabolic and hormonal changes, emphasizing the need to manage weight to prevent developmental delays
Cousminer et al. (2014) [53]	Genome-wide association study	6000 children and adolescents (aged 8–18 years)	Identified genetic loci associated with both higher BMI and earlier puberty (*p* < 0.05)	Shared genetic pathways may underlie both childhood obesity and early puberty
Deardorff et al. (2021) [48]	Longitudinal cohort study	700 Mexican–American boys and girls (aged 5–13 years) from the CHAMACOS cohort	Childhood overweight conditions and obesity were significantly associated with an earlier onset of pubertal markers (thelarche in girls, gonadarche in boys), with a stronger effect observed in girls (*p* < 0.01)	Childhood overweight conditions and obesity lead to earlier pubertal onset in Mexican–American children, with gender differences in the strength of associations
Euling et al. (2008) [42]	Review	Data from 17077 U.S. children and adolescents (aged 6–18) from 1940 to 1994	Pubertal timing shifted earlier over the decades especially in girls. Increased childhood obesity and environmental factors may play a role (*p* < 0.01)	Secular trends show earlier puberty onset over time likely due to a combination of genetic, nutritional, and environmental influences
Herman-Giddens et al. (2012) [46]	Cross-sectional study	4131 U.S. boys aged 6–16 years (mean age: 12 years)	Boys with higher BMI showed earlier pubertal onset (pubic hair and genital development) (*p* < 0.05)	Childhood obesity accelerates pubertal timing in boys
Kaplowitz (2008) [45]	Review	Various studies (no specific population size)	Increased adiposity impacted pubertal onset by influencing leptin and sex hormones (*p* < 0.01 in related studies)	Body fat influences pubertal timing through multiple mechanisms
Lee et al. (2010) [9]	Longitudinal cohort study	401 boys aged 5–12 years (U.S.)	Higher BMI was associated with earlier pubertal initiation, particularly in pubic hair and genital development (*p* < 0.01)	Childhood obesity is linked to earlier pubertal initiation in boys with stronger associations for higher BMI
Lee et al. (2016) [8]	Cross-sectional	1601 overweight and boys with obesity aged 6-16 years (U.S.)	Boys with obesity experienced delayed puberty, while overweight boys showed earlier onset compared to normal weight peers (*p* = 0.03)	Obesity may delay puberty onset, while overweight boys tend to have earlier puberty
Mohsenipour et al. (2022) [51]	Cross-sectional	168 children with obesity from Tehran, Iran, ages 7–14	Delayed puberty was significantly more common in boys with obesity than girls with obesity (*p* = 0.029)	Obesity may delay puberty in boys more frequently than in girls
Ong et al. (2006) [41]	Review	Various European population-based studies on timing and tempo of puberty (no specific population size)	Secular trends in Europe showed links between body size and earlier pubertal onset (*p* < 0.05)	Body size influences secular trends in pubertal timing in Europe
Reinehr and Roth (2019) [54]	Review	Various studies on obesity and puberty (no specific population size)	Leptin and other metabolic factors were involved in the relationship between obesity and puberty (*p* < 0.05 in several studies)	Obesity may influence puberty onset through metabolic and hormonal pathways

BMI = body mass index; HPG = hypothalamic–pituitary–gonadal.

**Table 2:** Main articles on the relationship between obesity and male fertility.

Reference	Type of Study	Study Population	Main Results	Conclusion
Salas-Huetos et al. (2021) [10]	Systematic review and meta-analysis	Various studies (60 for qualitative analysis, 28 for quantitative analysis) on association between adiposity, sperm quality, and reproductive hormones	Overweight and/or obesity were associated with low semen quality and altered reproductive hormones (*p* < 0.05)	Healthy body weight is important for sperm quality parameters and male fertility
AbbasiHormozi et al. (2023) [11]	Cross-sectional	40 healthy men, 40 men with obesity, 35 lean males with DM, 35 males with obesity and DM	Sperm parameters were significantly lower and leptin levels were significantly increased in cases compared to controls. Total testosterone and SHBG were significantly lower in men with obesity and DM. Insulin positively correlated to metabolic-associated indices and hsCRP, whereas it negatively correlated with sperm parameters	Obesity and diabetes are associated with metabolic changes, hormonal dysfunction, and inflammatory disturbance that may explain subfertility
La Vignera et al. (2019) [12]	Interventional study	Males with insulin-resistance and normogonadotropic idiopathic infertility receiving 150 units of FSH 3 times a week alone (n = 35, group A) or in association with slow-release metformin 500 mg/day (n = 35, group B)	Group B obtained higher sperm DNA fragmentation normalization rate (*p* = 0.03), sperm concentration, progressive motility, and morphology (*p* < 0.0001)	The addition of metformin in insulin-resistant infertile males improves the efficacy of FSH therapy
Tsilidis et al. (2013) [90]	Observational retrospective study	809 adult men	Higher testosterone was associated with lower CRP (*p* < 0.05), while higher estradiol correlated with higher CRP and WBC (*p* < 0.05). SHBG was inversely correlated to WBC (*p* = 0.04)	Higher androgen and lower estrogen correlate to anti-inflammatory markers in men
Yeap et al. (2014) [91]	Cross-sectional observational study	2143 men aged 17–97 years	Testosterone was inversely associated with metabolic syndrome score. In multivariable models, higher testosterone was associated with lower age, BMI, and CRP	Circulating androgens are more related to age and metabolic factors than cardiovascular or chronic disease
Pini et al. (2020) [93]	Observational study	5 men with no overt andrological diagnosis, analysis of sperm proteome (2034 proteins)	24 sperm proteins involved in inflammation, oxidative stress, DNA damage repair and sperm function were significantly (*p* < 0.05) less abundant in men with obesity compared with healthy weight controls	In men with obesity, oxidative stress and inflammation have negative impact on proteins involved in spermatogenesis, leading to subfertility
Thomas et al. (2013) [112]	Cross-sectional study	96 adult males stratified in normal weight, overweight, and obese groups	Adipokine levels were different in serum and seminal plasma. Higher BMI was associated with decreased seminal progranulin. Adiponectin and progranulin levels in seminal plasma correlated positively with sperm parameters (*p* < 0.05)	Adipokines influence sperm functionality
Moretti et al. (2014) [115]	Cross-sectional study	110 adult males, 47 with infertility and 63 without a history of infertility	Resistin concentration was higher in semen than in serum, had negative correlations with sperm motility, and positive correlations with apoptotic sperm and TNF-α and IL-6 levels. Cytokine levels were significantly higher in infertile patients compared with controls	Semen resistin may play a regulatory role in inflammation of the male reproductive system
Andersen et al. (2022) [128]	Randomized controlled trial	56 adult males (18–65 years) with obesity assigned to 8-week low-calorie diet followed by randomization to 52 weeks of either placebo, exercise training, liraglutide, or liraglutide combined with exercise training	Men lost on average 16.5 kg, which increased sperm concentration (*p* < 0.01) and sperm count (*p* < 0.01). These improvements were maintained for 52 weeks in men who maintained the weight loss	Sperm parameters improved after weight loss (both through exercise or liraglutide) in men with obesity
Raee et al. (2023) [13]	Cross-sectional cohort study	Semen analysis of 32 males with obesity and 32 normal-weight controls	Short telomere length was negatively correlated to BMI, sperm DNA fragmentation index, immature chromatin, and intracellular ROS levels in patients with obesity (*p* < 0.05). Obesity was associated with worse semen parameters and higher percentages of DNA fragmentation index, immature chromatin, apoptosis, and elevated ROS levels	Obesity is associated with sperm telomere shortening

DM = diabetes mellitus; SHBG = sex hormone-binding globulin; hsCRP = high-sensitivity C-reactive protein; WBC = white blood cells; GLP-1 = glucagon-like peptide 1; BMI = body mass index.
